# A Bioweapon or a Hoax? The Link Between Distinct Conspiracy Beliefs About the Coronavirus Disease (COVID-19) Outbreak and Pandemic Behavior

**DOI:** 10.1177/1948550620934692

**Published:** 2020-11

**Authors:** Roland Imhoff, Pia Lamberty

**Affiliations:** 1 Social and Legal Psychology, 9182 Johannes Gutenberg University, Mainz, Germany

**Keywords:** COVID-19, conspiracy beliefs, health behavior, coronavirus, pandemic, conspiracy mentality

## Abstract

During the coronavirus disease pandemic rising in 2020, governments and nongovernmental organizations across the globe have taken great efforts to curb the infection rate by promoting or legally prescribing behavior that can reduce the spread of the virus. At the same time, this pandemic has given rise to speculations and conspiracy theories. Conspiracy worldviews have been connected to refusal to trust science, the biomedical model of disease, and legal means of political engagement in previous research. In three studies from the United States (*N* = 220; *N* = 288) and the UK (*N* = 298), we went beyond this focus on a general conspiracy worldview and tested the idea that different forms of conspiracy beliefs despite being positively correlated have distinct behavioral implications. Whereas conspiracy beliefs describing the pandemic as a hoax were more strongly associated with reduced containment-related behavior, conspiracy beliefs about sinister forces purposefully creating the virus related to an increase in self-centered prepping behavior.

In November 2019, a 55-year-old man from the Hubei province in China was diagnosed with a new disease caused by a new virus SARS-CoV-2. In the beginning of 2020, the coronavirus pandemic has infected an enormous amount of people worldwide. Countries closed their borders, announced lockdowns, and asked people to follow protective measures against the new coronavirus, such as physical distancing and handwashing. Health systems were often not properly prepared to handle the influx of cases and arguably, the public information system was not prepared either. Already in February 2020, Dr. Tedros Adhanom Ghebreyesus, the Director General of the World Health Organization (WHO), warned that the world is “not just fighting an epidemic; we’re fighting an infodemic. Fake news spreads faster and more easily than this virus, and is just as dangerous” (WHO, 2020). A survey from mid-March 2020 conducted in the United States supported this notion: 42% of the U.S.-Americans have seen a lot or some news about the coronavirus outbreak which seemed completely made up (Mitchell & Oliphant, 2020). In the present article, we sought to better understand how such distorted beliefs about the coronavirus relate to the various ways to react to the pandemic. Specifically, we tested whether conspiracy beliefs claiming that the pandemic is a hoax are linked to a weaker support of containment-related behavior compared to perceiving the pandemic as human-made which should be linked to a stronger support of self-centered prepping behavior.

## The COVID-19 Pandemic and Conspiracy Beliefs

Past research shows that the increase in conspiracy theories during a pandemic is not a new phenomenon: Especially in times of crises, conspiracy thinking increases substantially (e.g., Van Prooijen & Douglas, 2017). For virtually all major events over the past decades, official versions of why these came about were confronted with various conspiracy allegations that proposed an explanation involving plots hatched in secret by powerful agents instead. This is also true for major outbreaks of diseases. A misinformation campaign run by the *Soviet* Committee for State Security claimed HIV to be a biological weapon developed by the United States (Geissler & Sprinkle, 2013), and the widespread belief that AIDS is a conspiracy to kill Black people has a direct impact on prevention behavior (e.g., using condoms or pre-exposure prophylaxis; e.g., Bogart & Thorburn, 2005; Bogart et al., 2010). During the Zika virus outbreak of 2015–2016, there were speculations that the virus was caused by genetically modified mosquitoes or used by the governments to kill people on purpose (e.g., Klofstad et al., 2019).

Events of such magnitude beg an explanation of comparable magnitude (Leman & Cinnirella, 2007). Providing explanations is psychologically advantageous for several reasons, with one sticking out in the previous literature: granting an illusion of control. Considering this reasoning, it is not surprising that a lack of control has been identified as one of the key drivers of conspiracy beliefs. When people are not able to gain control in the real world, they compensate for this lack by perceiving patterns—even if they are an illusion (e.g., Douglas et al., 2017). The current coronavirus crisis is an almost ideal breeding ground for conspiracy thinking (Van Bavel et al., 2020), as there is no easily comprehensible mechanistic explanation of the disease, it is an event of massive scale, it affects people’s life globally and leaves them with lots of uncertainty.

Such conspiracy beliefs might potentially even be palliative in giving people back at least a sense of control. Nevertheless, so we argue, there are real dangers in such conspiracy theories as they might motivate problematic behavior in the current crisis. During the coronavirus pandemic, many scientists, specifically epidemiologists and physicians, have been the most articulate voices in making recommendations on how to “flatten the curve” and slow down the infections. *Conspiracy mentality*, however, a generalized belief that powerful forces operate in secret to rule the world (Imhoff & Bruder, 2014), has been connected to distrust in both science in general (as it is perceived as high power; Imhoff et al., 2018) and the biomedical system more specifically (for the same reason; Galliford & Furnham, 2017; Lamberty & Imhoff, 2018; Oliver & Wood, 2014). Thus, people who endorse a conspiracy worldview are particularly unlikely to trust the expert recommendations aimed at reducing infection rates.

Whereas most people use information about what others do as a cue to how to behave themselves and thus are more likely to show conformity and follow (descriptive) social norms, there are some exceptions to this rule. Specifically, people high in a *need for uniqueness*, for whom it is of great importance to stick out from the crowd, are intentionally trying to not do or say what the majority of people say or do (Imhoff & Erb, 2009). This is relevant, as endorsement of conspiracy beliefs has been associated with an increased need for uniqueness in both correlational and experimental studies (Imhoff & Lamberty, 2017; Lantian et al., 2017). Thus, conspiracy believers are less likely than others to comply with descriptive social norms. Additionally, a conspiracy-prone worldview does not only reduce trust in official versions and adherence to norms but is also linked to a stronger acceptance of violence (Rees & Lamberty, 2019). Conspiracy worldviews also make it more plausible to engage in illegal, nonnormative forms of action to reach one’s goals, as people who imagined seeing the world as people high in conspiracy mentality saw it as more defensible to use force and other illegal means to pursue one’s political goals (Imhoff et al., 2020).

## Distinct Effects of Different Conspiracy Beliefs on Behavior

Many of the above-cited findings rely on associations between certain attitudes or behavioral intentions with a generalized conspiracy worldview (e.g., Imhoff & Bruder, 2014; Moscovici, 1987; Popper, 2006). The reasoning behind this relies on the robust finding that content-wise even completely unrelated conspiracy beliefs are so highly intercorrelated that they typically load on one factor (Bruder et al., 2013; Swami et al., 2011) and are thus often understood as specific expressions of a generalized mindset or political attitude (Imhoff & Lamberty, 2018). This goes as far as logically incompatible conspiracy theories correlating positively (Wood et al., 2012).

Although this general pattern seems to be one of the most robust findings in the psychology of conspiracy theories, in the current COVID-19 situation, two popular conspiracy theories did not only seem to be logically incompatible but might be related to different behaviors as well. While many people played down the danger of COVID-19, calling it no worse than a flu, and suspected others to purposefully claim otherwise for their own advantage (e.g., hurting national economies, passing unpopular/restricting laws), others painted an even more drastic picture by claiming that the new coronavirus had not evolved by mutation (Andersen et al., 2020) but had been intentionally manufactured and purposefully spread as a bioweapon for political or economic gains. A survey conducted in the beginning of March 2020 in the United States supported the notion to differentiate between the two types of conspiracy beliefs: The results showed that 49% claimed that the coronavirus is a man-made epidemic. In contrast, 44% thought that the threat of the coronavirus is being exaggerated for political reasons and 13% were convinced that the coronavirus is a hoax (Frankovic, 2020).

The denial or downplay of the danger of an illness should directly affect the risk assessment of a person, and the perception of illness-related risks influences in turn directly health-promoting self-care behavior (e.g., Ferrer & Klein, 2015; Rosenstock, 1974). A higher perceived risk is, for example, associated with a greater likelihood to engage in protective behavior (Brewer et al., 2004). Therefore, if people believe that the COVID-19 pandemic is a hoax or exaggerated by the government, they should be less likely to follow official recommendations such as handwashing and social distancing (see also Stanley et al., 2020).

On the other hand, many people are convinced that the virus was created in a lab—either accidentally or intentionally to “reduce the population” as a secret plan of a so-called “new world order.” People who hold these kinds of beliefs should be less likely to underestimate the severity of the coronavirus outbreak since they perceive it as an attack of governments or secret services against “the people.” As a consequence, these people should not follow the recommended behavior of the institutions that they suspect of plotting a conspiracy (i.e., governments, WHO, health care providers) and might instead follow their own policies of protection against the pandemic (e.g., alternative medicine, weapons, hoarding).

## The Present Research

Conspiracy theories that suggest that the coronavirus pandemic is a hoax are expected to primarily be related to refusal to engage in containment-related behavior (e.g., hygiene, physical distancing). Conspiracy theories that describe SARS-CoV-2 as a human-manufactured virus are expected to mainly relate to more self-centered prepping behavior (e.g., alternative remedies, hoarding). Despite these divergent associations (and the logical inconsistency), we expect both conspiracy beliefs to be positively correlated and positively correlated with conspiracy mentality. All materials and data (including the Supplemental Material) are available at https://osf.io/6p8tv.

## Study 1

Study 1 was an *ad hoc* inclusion of relevant measures in a planned data collection for a retest of a scale tapping into maintenance motivation unconnected to the current article (Ecker et al., 2020). With the current research question in mind, we also added (in that order) questions about perceived threat by COVID-19, pandemic-related behaviors, endorsement of conspiracy beliefs, and a measure of general conspiracy mentality. We expected conspiracy beliefs that COVID-19 is a hoax or its relevance exaggerated to be associated with hesitancy to follow official recommendations but conspiracy beliefs stating that SARS-CoV-2 was human-made to be associated with increased tendencies to engage in prepping behavior. Despite these dissociated associations, we did expect to replicate the well-established finding that the two are positively correlated. Data collection took place between March 20 and March 23, 2020.

## Method

### Participants

We invited 280 *Mechanical Turk* workers who had participated (and passed an attention check) in a study run two weeks earlier. Of these, 237 accepted the invitation within the first 3 days (over which participation dropped continuously and prompted the decision to terminate data collection: Day 1: 212, Day 2: 14, Day 3: 6, Day 4: 5) and participated in the current study, but *n* = 17 failed an attention check (“To indicate that you read this item carefully, please mark the lowest rating”) and were thus excluded from the sample. This left a final sample of *N* = 220 (118 men, 97 women, 5 other; *M*_age_ = 40.18, *SD*_age_ = 12.33; 79% identified as White, 9% as Black/African American, 9% as Asian) with a median annual income of US$40,000, implying 80% power to detect correlations of ρ = .17 or higher.

### Measures

#### COVID-19 conspiracy beliefs

We created two sets of 3 items (one reverse-coded) to tap into the two most prevalent conspiracy beliefs. To tap into the idea that it is a harmless virus that receives overblown attention for personal benefit of a few people (COVID-19 hoax), we asked participants for their agreement with the following statements: “The virus is intentionally presented as dangerous in order to mislead the public,” “Experts intentionally mislead us for their own benefit, even though the virus is not worse than a flu,” and “We should believe experts when they say that the virus is dangerous” (reverse-coded). The (logically incompatible) notion that the virus was purposefully created for the personal benefit of a few people (SARS-CoV-2 human-made) was assessed by asking for agreement with the following propositions: “Corona was intentionally brought into the world to reduce the population,” “Dark forces want to use the virus to rule the world,” and “I think it’s nonsense that the virus was created in a laboratory” (reverse-coded). All six statements were completed on a scale ranging from 1 (*strongly disagree*) to 7 (*strongly agree*).

#### Pandemic-related behavior

To tap into respondents’ self-reported pandemic-related behavior, we asked them to report for 18 possibilities as to what extent they behaved this way from 1 (*never*) to 7 (*always/strongly*). Specifically, participants were informed that “people have reacted differently to the emergence of the new coronavirus. Below, we ask you to indicate for each of several behaviors as to what extent you have implemented this as part of your reaction to the coronavirus.” These behavior options were either containment-related (e.g., increased hygiene behavior, keeping physical distance from others) or self-centered prepping behavior (e.g., hoarding everyday goods, relying on “alternative” sources of information or remedies; see Table 1 for the full list as well as factor loadings).

**Table 1.** Factor Loadings for Exploratory Factor Analyses of Self-Reported COVID-19-Related Behaviors.

Pandemic-related Behaviors	Study 1 (United States)		Study 2a (United States)		Study 2b (UK)	
1. Disinfecting hands after being outside		.783		.704		.375
2. Avoiding social contacts		.744		.716		.700
3. Washing hands after being outside		.740		.715		.715
4. Avoiding crowds		.725		.840		.643
5. Not touching the face while being outside		.660		.741		.477
6. Staying at home in quarantine		.614		.591		.448
7. Stocking up on food	.457	.558	—	—	—	—
8. Stocking up on sanitary items	.492	.541	.485	.317	.442	
9. Buying weapons for defense and security purposes	.702		.751		.396	
10. Stocking up on petrol and oil	.723		.812		.372	
11. Buying equipment for water storage and water purification	.685		.810		.530	
12. Withdrawing available cash from my bank account	.662		.777		.479	
13. Wearing protective face masks out of the house	.625		.729		.576	
14. Invest in stock market	.474		(.690)			
15. Using alternative remedies like homeopathy or essential oils	.506		.766		.479	
16. Searching information by alternative media online	.530	.312	.483		.418	
17. Spreading information online	.519		—	—	—	—
Eigenvalue (% of variance)	2.54 (14.09)	4.98 (27.66)	4.88 (32.51)	3.06 (20.37)	1.71 (11.39)	2.32 (15.46)

*Note*. *N_Study1_* = 220; *N_Study2a_* = 288; *N_Study2B=_*298. All analyses conducted with promax rotation and the kappa parameter of 4, as the generally recommended default (Hendrickson & White, 1964). Loadings under .30 are suppressed. One item included in Study loaded ∼ .35 on both factors and was thus excluded (“Search information by official virologists online”).

#### COVID-19 threat perception

We asked participants with 4 items how strongly they felt affected by the outbreak (“To what extent are you currently worried about the spread of coronavirus?” “To what extent are you currently personally affected by the spread of coronavirus?” “To what extent do you currently feel threatened by the spread of coronavirus?” and “To what extent are you at risk for COVID-19 complications?”) on a scale from 1 (*not at all*) to 7 (*very much*).

#### Additional variables

For exploratory purposes, we also included the 12-item Conspiracy Mentality Scale (Imhoff & Bruder, 2014; e.g., “There are secret organizations that have great influence on political decisions.” 7-point scale from *strongly disagree* to *strongly agree*). Additionally, 1 item tapped into self-reported political orientation on a scale from 1 (*extremely liberal*) to 7 (*extremely conservative*).

## Results

All scales proved to be satisfactorily reliable (Table 2). The *exploratory factor analysis* of pandemic-related behavior clearly suggested a two-factor solution. Only 2 items on hoarding behavior (stocking up on food and sanitary items) exhibited double loadings (see Table 1). This may be due to the fact that the “hoarding” of such goods (which was discussed as an overreaction and primarily problematic) had created a situation where (a) these goods were indeed becoming scarce and (b) to avoid physical contact; less frequent grocery shopping (and thus “stocking up”) was becoming instrumental. We thus excluded these 2 items and averaged the others to composite scales of containment-related behavior and self-centered prepping behavior.

**Table 2.** Intercorrelations of the Key Variables in Study 1.

Pandemic-related Behaviors	*M*	*SD*	α	1.	2.	3.	4.	5.	6.
1. COVID-19 Hoax	2.08	1.35	.848						
2. SARS-Cov-2 Human-Made	2.46	1.45	.672	**.511**					
3. Containment-related behavior	5.87	1.15	.859	**−.356**	−.123				
4. Self-centered prepping behavior	2.36	1.20	.827	**.256**	**.342**	**.227**			
5. COVID-19 Threat	4.35	1.41	.820	**−.305**	−.038	**.429**	**.229**		
6. Conspiracy Mentality	4.17	1.35	.925	**.357**	**.523**	.042	**.236**	.046	
7. Political Orientation	3.29	1.66	—	**.261**	**.320**	−.014	**.202**	—.055	.063

*Note*. *N* = 220. Bonferroni-adjusting for all 28 correlations yields correlations ≥ .210 significant (*p* < .00178), printed in bold.

As expected, the two specific conspiracy beliefs were highly correlated and associated with conspiracy mentality as a general mindset. People who believed that the pandemic was a hoax were more likely to perceive the pandemic as less threatening, while there was no significant link between assuming that the virus was human-made and threat perception (Table 2). To test our focal hypothesis that the two distinct conspiracy beliefs were distinctly associated with recommended and nonrecommended reactions to the coronavirus pandemic, and whether this would hold above and beyond effects of political orientation, we regressed the respective self-reported behaviors on the two conspiracy belief scales and added political orientation.^1^ In line with our predictions, believing that COVID-19 was a hoax was a strong negative prediction of containment-related behaviors such as handwashing and keeping physical distance, *B* = −0.345, *SE* = 0.063, *p* < .001, whereas believing in a human origin of the coronavirus was not, *B* = 0.049, *SE* = 0.060, *p* = .413. Self-reported conservatism had no prediction above and beyond these, *B =* .050, *SE =* .047, *p = .*286. The local effect size of COVID-19 hoax above and beyond the other predictors was thus *f*^2^ = .133.

For self-centered prepping behavior, the pattern was less clear-cut as it was uniquely associated (as expected) with conspiracy beliefs about human creation of the coronavirus, *B* = 0.217, *SE* = 0.063, *p* = .001, but not with the idea that COVID-19 is a hoax, *B* = 0.087, *SE* = 0.066, *p* = .187, or conservatism, *B* = 0.067, *SE* = 0.049, *p* = .173. Thus, even claiming that COVID-19 was not worse than a common flu was associated with self-reports of behavior characterized as overreacting (e.g., hoarding), but SARS-CoV-2 human-made still had a substantial effect, *f*^2^ = .068. Exploratory analyses show that these associations are generally stronger, the more threatened people felt by the virus (see the Supplemental Material).

## Discussion

Study 1 provides first evidence that—although there is a certain overlap of constructs—different conspiracy theories are associated with different types of self-reported behavior. While people who belittle the risk of COVID-19 are less likely to follow official recommendations, people who believe that the virus originated in a laboratory are more likely to prepare for worst-case scenarios. Intriguingly, these strong reactions were independently related also to the endorsement that COVID-19 is no worse than a flu, particularly for those who feel strongly threatened by it. This finding raises some doubt as to what extent these respondents were actually fully convinced of their own opinion that it was an overblown but actually harmless disease. In light of the purely exploratory nature, the ad hoc construction of scales and the scarcity of control variables, we conducted a set of replications and extensions in two different national contexts to bolster our findings’ generalizability.

## Study 2

The main aim of Study 2 was to replicate the results of Study 1 in two national contexts: the United States and the UK. For this purpose, the double-loading behaviors were removed, and we aimed to show the specific influence of conspiracy beliefs as distinguished from other constructs. Most notably, people’s reactions to the pandemic have been associated with political ideology. While we controlled for this in Study 1, it was a mere 1-item measure with unclear reliability. We thus included 2 multi-item scales of well-established constructs of political ideology: *right-wing authoritarianism* (RWA) and *social dominance orientation* (SDO). Despite being more specific and more reliable measures of overall political orientation, both also entail specific aspects. People high in RWA tend to follow norms and be obedient to authorities, allowing the prediction of greater adherence to official recommendations. High SDO, on the other hand, reflects a belief system of a “dog-eat-dog” world (Pratto et al., 1994). Therefore, people with a pronounced SDO should rather follow prepping behavior that benefits themselves. We also added the *Big Five* personality traits as control variables frequently associated with health behavior (e.g., Atherton et al., 2014). All data were collected on March 25. The night before data collection was planned to start in the United States and the UK, the UK Prime Minister Johnson declared a lockdown. For this reason, we had to adjust the wordings of the items inquired about *past* behavior. For the UK, we also included an exploratory measure of intentions to comply with this lockdown. We partly deviated from our preregistration (https://aspredicted.org/5nx8k.pdf), as we detail in Supplementary Table S1.

## Method

### Participants

To achieve 80% power to observe the smaller of the two effects found in Study 1, only 110 participants were required. In light of general recommendations for robust correlational estimates, we aimed for a sample of *N* = 300 in each substudy, which equipped us with 1 − β > .99 for both effects. For Study 2a, we invited 300 U.S.-based *MTurk* workers (82% identified as White, 11% as Black/African American), out of which 12 recommended to not use their data, leaving a final sample of *N* = 288 (169 men, 117 women; *M*_age_ = 36.60, *SD*_age_ = 11.16). For Study 2b, the same number of UK-based participants was recruited via *Prolific Academic*. Only two participants recommended to not use their data, resulting in a final sample of *N* = 298 (123 men, 172 women; *M*_age_ = 37.29, *SD*_age_ = 12.79; we did not record data on ethnicity).

### Measures

#### Measures for confirmatory analyses

All measures for our confirmatory analyses (two conspiracy beliefs, pandemic behaviors) were taken from Study 1 with the exception that three behaviors were deleted from the Self-Reported Behavior Scale (items 7, 9, and 18 in Table 1).

#### Measures for exploratory analyses

In addition, the single political orientation item and the scale tapping into feelings of threat also copied from Study 1, we specifically asked whether respondents or someone they knew had been tested positive for COVID-19 (“Have you or someone you know been tested positive for COVID-19?”). Only in the UK version of the survey, we included a scale in response to the declared lockdown effective from that day on (“Now that there is a lockdown, please let us know about the behavior you expect to show.”). We designed six questions tapping into people’s intentions to disregard the lockdown regulation (e.g., “hang out in groups with friends in public places” and “go directly home from work/grocery shopping without seeing anyone” [reverse-coded]), which were completed on the same scale as the COVID-19-related actions. As additional control variables, we added a measure of RWA (Nießen et al., 2019; e.g., “We need strong leaders so that we can live safely in society.”; item order randomized), of SDO (Ho et al., 2015; e.g., “Some groups of people are simply inferior to other groups.”; item order randomized), and a short 10-item scale to tap into the Big Five personality facets (Rammstedt et al., 2014; fixed item order).

## Results

### Descriptive Analyses

The majority of our samples did not know persons tested positive for COVID-19 (United States: 87%; UK: 92%). Nevertheless, 16 U.S.-based participants (5%) had been tested positive themselves and another 11 (4%) shared a home with a person tested positive (*n* = 2 in the UK sample). The proportion of participants with a positive case in their extended surroundings was larger in the UK sample (*n* = 20; 7%) than the U.S. sample (*n* = 10; 3%). We ran initial exploratory factor analyses (EFAs) on the pandemic-related behaviors in both substudies to include only behaviors that loaded at least .30 on one but only one of the two factors. All items but 1 met this criterion. Investing in the stock market, however, showed strong loading on the nonrecommendable factor in the United States but no loading at all in the UK. Building composite scores of self-centered prepping behavior with and without this item yielded highly correlated measures in both samples (*r* = .99) and did not yield any difference on any of the central analyses. We thus kept the item in both substudies to enhance comparability. For descriptive purposes, Table 3 shows all measured variables and their intercorrelations.

**Table 3.** Intercorrelations of the Key Variables in Study 2a and Study 2b.

Pandemic-related Behaviors	Study 2a (United States)			Study 2b (UK)			1.	2.	3.	4.	5.	6.	7.	8.	9.	10.	11.	12.	13.	14.	15.
	*M*	*SD*	α	*M*	*SD*	α															
1. COVID-19 Hoax	2.51	1.59	0.81	1.74	1.03	0.81		**.693**	**−.524**	**.554**	—	−.082	**.407**	**.415**	**.418**	**.594**	**−.315**	**−.340**	.139	**−.317**	.164
2. SARS-Cov-2 Human-Made	2.87	1.57	0.68	2.35	1.36	0.74	**.509**		**−.307**	**.605**	—	.111	**.570**	**.439**	**.473**	**.509**	**−.309**	**−.264**	.113	**−.326**	.076
3. Containment-related behavior	5.81	1.18	0.86	5.84	0.92	0.68	−.154	.014		−.133	—	**.367**	−.106	−.076	−.078	**−.309**	.174	**.328**	−.111	**.209**	−.071
4. Self-centered prepping behavior	3.08	1.54	0.90	1.90	0.73	0.66	.184	**.304**	**.217**		—	**.447**	**.414**	**.340**	**.601**	**.455**	**−.340**	**−.372**	.174	−.219	.149
5. Non-compliance with lockdown	—	—	—	1.51	0.62	0.63	**.276**	.066	**−.402**	.051		—	—	—	—	—	—	—	—	—	
6. COVID-19 Threat	4.62	1.36	0.82	4.60	1.10	0.68	**−.254**	.023	**.253**	.131	**−.252**		.165	.099	**.312**	.032	−.085	−.111	.044	−.037	.165
7. Conspiracy Mentality	4.12	1.26	0.90	4.09	1.20	0.92	**.365**	**.506**	.017	**.241**	.036	.023		**.296**	**.353**	**.321**	−.097	−.122	−.040	**−.348**	.137
8. Political Orientation	3.57	1.91	—	3.52	1.49	—	.194	**.212**	.073	**.266**	.054	−.035	.092		**.506**	**.545**	**−.269**	−.144	.037	−.191	.121
9. Right-Wing Authoritarianism	2.73	0.95	0.91	2.90	0.74	0.84	.159	**.207**	.016	.157	−.001	.020	.082	**.433**		**.557**	**−.412**	**−.270**	.171	−.061	.104
10. Social Dominance Orientation	2.68	1.41	0.91	2.50	1.14	0.88	**.273**	**.233**	−.145	**.279**	.177	−.042	.172	**.590**	**.464**		**−.386**	**−.331**	.165	**−.321**	.189
11. Openness (Big 5)	3.59	0.96	0.45	3.56	0.97	0.52	−.082	−.026	.085	.051	−.044	.087	.067	**−.244**	−.143	−.183		**.301**	.053	.196	−.106
12. Conscientiousness (Big 5)	3.9	0.92	0.54	3.87	0.82	0.59	−.031	.061	**.223**	.101	−.092	.174	−.071	.158	**.233**	.001	159		.074	**.303**	**−.405**
13. Extraversion (Big 5)	2.6	1.01	0.54	2.77	0.96	0.69	−.097	.011	.160	.060	−.017	.062	−.063	−.037	.047	−.076	.084	**.205**		.139	**−.285**
14. Agreeableness (Big 5)	3.43	0.97	0.38	3.47	0.87	0.40	.039	−.051	.017	−.102	.043	−.014	−.165	−.064	.055	**−.209**	−.036	**.280**	.082		**−.333**
15. Neuroticism (Big 5)	2.62	1.08	0.68	2.98	1.04	0.64	−.047	.046	−.089	−.088	−.127	.114	.054	−.146	−.090	−.107	.135	−.177	**−.246**	−.150	

*Note*. Descriptives and correlations for US (N = 288; correlations above the diagonal) and UK (N = 298; correlations below the diagonal) sample. Correlation coefficient significant at Bonferroni-corrected level for 105 bivariate correlations (p=.000476) are printed in bold.

### Confirmatory Analyses

As predicted, both conspiracy beliefs were positively correlated as well as correlated with a general conspiracy mentality in both samples (Table 3). To test our central predictions that doubting either the seriousness of COVID-19 (hoax) or the natural origin of the coronavirus (human-made) had distinct implications for self-reported behavior, we regressed recommended and self-centered prepping behavior on both conspiracy beliefs simultaneously.

As predicted, containment-related behavior was negatively predicted by believing that COVID-19 was a hoax, *B* = −0.448, *SE* = 0.052, *p* < .001, but not by believing the coronavirus was human-made, *B =* 0.080, *SE =* 0.052, *p* = .129, in the U.S. sample, *f*^2^ = .260, as well as the UK sample, even if less pronouncedly, *f*^2^ = .036 (*B = −*0.196, *SE =* 0.060, *p* = .001 for *hoax*; *B =* 0.085, *SE =* 0.045, *p =* .060 for *human-made*). To test whether these results were significant, we subjected the difference in standardized regression coefficients to a significance test following Cohen and colleagues (2003; appendix 2.1). Hoax beliefs were stronger predictors than human-made beliefs both in the United States, Δβ = .707, *t*(285) = 12.98, *p* < .001, and the UK, Δβ = .343, *t*(295) = 5.21, *p* < .001.

On the contrary, self-centered prepping behavior in the U.S. sample was strongly associated with believing in the human-made origin of the coronavirus, *B* = 0.412, *SE* = 0.0162, *p* < .001, *f*^2^= .157, albeit also (seemingly paradoxically) with the belief in a COVID-19 hoax, *B* = 0.252, *SE* = 0.062, *p* < .001. Self-centered prepping behavior was indeed more strongly associated with human-made beliefs than with hoax beliefs, Δβ = .162, *t*(285) = 3.25, *p* = .001. Both remained significant predictors when controlling for all other variables (Supplemental Table S3). In the UK sample, the effects of the two conspiracy beliefs were similar, albeit both smaller, yielding a significant relation to human-made beliefs, *B* = 0.153, *SE* = 0.035, *p* < .001, *f*^2^ = .066, but not the same (paradoxical) one with hoax beliefs, *B* = 0.028, *SE* = 0.046, *p* = .538. This difference in standardized βs, Δβ = *−*.244, was again different from zero, *t*(295) = 3.82, *p* < .001.

### Exploratory Analyses

We had based our central hypotheses on the simple comparison between the predictive validity of both conspiracy beliefs and this was supported by the data. At the same time, it might be that these correlations are spurious due to shared influence of third variables (e.g., political ideology). To control for the respective unique associations, in a second step we added measures of political ideology in a stepwise procedure (to avoid multicollinearity and suppression issues due to their high intercorrelation) and added the personality measures, age, and COVID-19 threat in a third step. The full regression tables are available in the Supplemental Material (Tables S2–S6); importantly, however, controlling for all these variables altered the results only in one regard: the unique prediction of containment-related behavior by hoax beliefs was no longer significant, but political orientation and SDO, both uncorrelated at the level or zero order correlations, became significant predictor, albeit in different directions (Table S4).

In the preregistration, we had mentioned the inclusion of a measure of compliance with lockdown regulations in the UK sample but failed to specify predictions. Following from the proximity to containment-related behavior, we tested whether hoax beliefs would not only relate to past containment-related behavior but also to future expectation of complying with (at that time very new) lockdown regulations. We also thus applied the same analytical procedure to the intended noncompliance with lockdown regulations. Mirroring the results for containment-related behavior, this noncompliance was associated with hoax beliefs, *B =* .197, *SE =* .039, *p* < .001, *f*^2^ = .09 but not human-made beliefs, *B = −*.046, *SE =* .029, *p = .*120, Δβ = .429, *t*(295) = 6.69, *p* < .001 (Table S6).

## Discussion

Overall, Study 2 replicated Study 1—both in the United States and the UK. The belief that COVID-19 is a hoax was particularly associated with a reduced containment-related behavior. Conversely, a stronger belief that the virus originated in the laboratory was associated with a stronger advocacy of self-centered prepping behaviors. These effects remained stable even when controlling for other relevant variables. Overall, the effects were substantially weaker for the UK than in the United States, even if they followed the same patterns. Although the primary function of the other included variables was to critically test the unique variance of conspiracy beliefs, some patterns seem noteworthy. First, feeling threatened by the COVID-19 disease was associated with greater conformity with containment-related behavior but also more pronounced self-centered prepping behaviors. Pronounced threat thus does not operate as a functional mechanism to enhance (only) containment-related behavior. Its association with self-centered prepping behavior was—at least in the United States—particularly pronounced for those endorsing either kind of conspiracy beliefs. As would be expected, greater compliance with official recommendation was also related to conscientiousness, a personality trait associated with rule compliance and orderliness.

## General Discussion

We observed similarly problematic correlates of two distinct conspiracy beliefs concerning the coronavirus pandemic. Depending on whether COVID-19 was believed to be a hoax or the SARS-CoV-2 human-made, participants indicated less compliance with self-reported infection-reducing, containment-related behavior and more engagement in self-reported self-centered prepping behavior targeting not a reduction of the infection rate but personal benefits in the crisis. Although these associations seem relatively straightforward, it is important to note that previous research has pointed to the danger of conspiracy beliefs but has dedicated less attention to potentially distinct relations of different kinds of conspiracy beliefs. These distinct associations notwithstanding, our results also provide strong support for the general notion that even logically incompatible conspiracy beliefs show a high correlation and are positively associated with a general mindset of conspiracy mentality.

Adding to the robustness of the findings, another study conducted within the German context (reported in the Supplemental Material) closely replicated this general pattern. This seems noteworthy as another data set from the German context failed to find strong relations between conspiracy endorsement and hygiene measures (Pummerer & Sassenberg, 2020).

### Limitations and Further Research

As arguably the most important limitation of our research, all findings are cross-sectional correlations and thus mute with regard to causality. Although it seems plausible that people adapt their behavior according to how they see and perceive the world, it is also conceivable that people behave in a certain way (for no or other reasons) and adapt their worldview as a justification after the fact. Another clear limitation is that these studies were conducted in a time of rapidly changing world events and thus might not have undergone the amount of planning and detailed preregistration as generally desirable for any kind of research question (Scheel, 2020). Applying a stricter α level (e.g., *p = .*005; for a discussion, see Benjamin et al., 2018) would yield the negative impact of hoax belief on self-reported containment-related behavior in the UK nonsignificant after including all control variables. Trusting in the readers’ intuition to interpret the results, we refrain here from forcing a binary significant–nonsignificant decision on these data but merely point to the substantially weaker data pattern in the UK compared to the United States.

## Supplemental Material

Supplemental Material, Corona_Conspiracy_Paper_ImhoffLamberty_suppl - A Bioweapon or a Hoax? The Link Between Distinct Conspiracy Beliefs About the Coronavirus Disease (COVID-19) Outbreak and Pandemic Behavior Click here for additional data file. Supplemental Material, Corona_Conspiracy_Paper_ImhoffLamberty_suppl for A Bioweapon or a Hoax? The Link Between Distinct Conspiracy Beliefs About the Coronavirus Disease (COVID-19) Outbreak and Pandemic Behavior by Roland Imhoff and Pia Lamberty in Social Psychological and Personality Science

## Note

1. As income might be a proxy for education which has been associated with conspiracy beliefs (van Prooijen, 2017), we also controlled for that but found no association with either criterion, *p*s > .671.

**Supplemental Material:** The supplemental material is available in the online version of the article.
