# Supplementary material for: A Bioweapon or a Hoax? The Link Between Distinct Conspiracy Beliefs About the Coronavirus Disease (COVID-19) Outbreak and Pandemic Behavior
Source: Soc Psychol Personal Sci. 2020 Nov;11(8):1110–8. doi: 10.1177/1948550620934692 (PMC7342934; doi:10.1177/1948550620934692)
Supplement: Supplemental Material, Corona_Conspiracy_Paper_ImhoffLamberty_suppl - A Bioweapon or a Hoax? The Link Between Distinct Conspiracy Beliefs About the Coronavirus Disease (COVID-19) Outbreak and Pandemic Behavior [file Corona_Conspiracy_Paper_ImhoffLamberty_suppl.pdf]

Supplemental Materials to

Imhoff, R., & Lamberty, P. (2020, April 14). A bioweapon or a hoax? The link between distinct conspiracy beliefs about the Coronavirus disease (COVID-19) outbreak and pandemic behavior. *Social Psychological and Personality Science*.

Supplement 1: Exploratory Moderation by Perceived Threat

In all three samples, we conducted exploratory analyses to test whether the relation between conspiracy beliefs and (non-recommended) self-centered prepping behavior were generally stronger, the more threatened people felt by the virus. Specifically, in Study 1 adding an interaction term of the endorsement of one of the conspiracy beliefs and the perceived threat by the coronavirus, led to significant interaction terms and increases in explained variance in the extent of non-recommendable behavior, $B = 0.297$, $SE = 0.064$, $p < .001$, $\Delta R^2 = .074$, p < .001 for hoax, $B = 0.183$, $SE = 0.068$, $p = .008$, $\Delta R^2 = .027$, p = .008 for human-made, in the form that the association between conspiracy belief and self-centered prepping behavior generally became stronger, the more threatened people felt (Figure 1). A higher order model additionally suggested a three-way interaction (see supplement), but we refrained from putting too much interpretative weight on it before replicating these interactions.

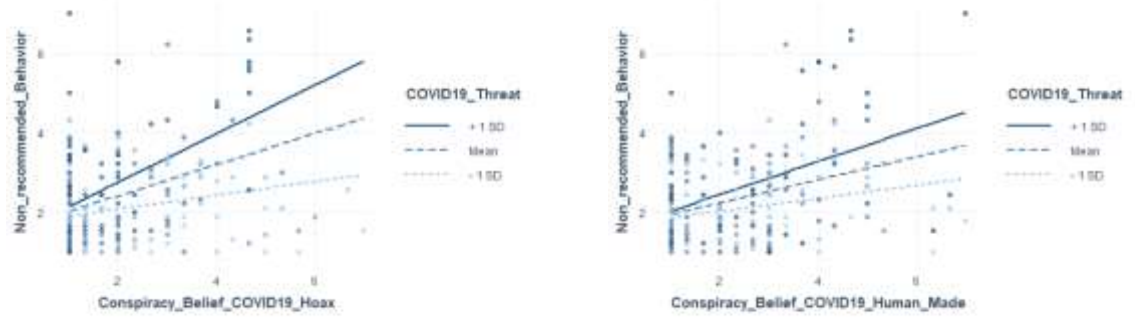

Figure 1. Self-centered prepping behavior as a function of distinct conspiracy beliefs moderated by perceived threat in Study 1.

We tested in Study 2 whether this moderation, with COVID-19 threat amplifying the relation between the respective conspiracy beliefs and self-centered prepping behavior, would replicate. In short, the pattern replicated for both interactions (but not the three-way interaction) in the US sample (B = 0.275, SE = 0.052, p < .001, ΔR^2 = .041, p < .001 for hoax; B = 0.198, SE = 0.060, p < .001, ΔR^2 = .018, p = .001 for human-made), but not in the UK sample (for both $\Delta R^2 \leq .002$, ps $\geq$ .490). Thus, the effect seems to be reliable albeit specific to the context of the USA (Figure 2).

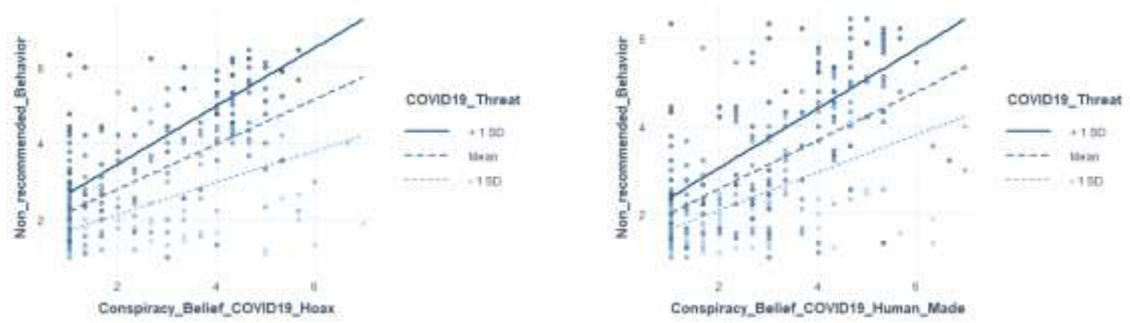

Figure 2. Self-centered prepping behavior as a function of distinct conspiracy beliefs moderated by perceived threat in Study 2a (USA).

Supplement 2: Detailed explanation and deviation from pre-registration in Study 2

Due to the rushed nature of data collection in the given social context our pre-registration for Study 2 was not as detailed enough as desirable. In addition, there have been some (data-independent) changes to the pre-registered plan. Below we thus provide more detail.

Table.S1

WORDING IN PRE-REGISTRATION	*EXPLANATION (+ POTENTIAL DEVIATION)*
**2) What's the main question being asked or hypothesis being tested in this study?** Testing the relation between two different COVID-19-related conspiracy theories and COVID-related behavior. Conspiracies that COVID is a hoax are expected to be primarily related to refusal ton engage in recommended actions (hygiene, physical distancing). Conspiracy theories that COIVD-19 is a human-manufactured virus are expected to mainly predict non-recommended actions (alternative remedies, hamstering). Despite these divergent predictions (and the logical inconsistency), we expect both CTs to be positively correlated and also correlated with conspiracy mentality.	*At the time of the pre-registration, the most sense we could make of the factor structure in Study 1 was that one factor included all the actions recommended by the WHO and national health agencies at that time, whereas the others mostly included behaviors that were depicted as problematic by these same institutions. It therefore seemed intuitive to think of them as "recommended" and "non-recommended" and this is also the terminology used in the syntax:*<br><br>`compute action_reco = mean (COVID_reactions_1, COVID_reactions_2, COVID_reactions_3 , COVID_reactions_5, COVID_reactions_6, COVID_reactions_8).`<br>`compute action_non = mean (COVID_reactions_4, COVID_reactions_7, COVID_reactions_9, COVID_reactions_10, COVID_reactions_11, COVID_reactions_12, COVID_reactions_13, COVID_reactions_14, COVID_reactions_15).`<br><br>*After collecting data for Study 2, however, it dawned on us that there is a deeper, a psychological difference between them that seem much more interesting to us. While the former are mostly solidarity-oriented in the sense of breaking infectious cycles and containing the spread, the latter are just about protecting oneself and getting through the crises as unharmed as possible.*<br><br>*The hypotheses were tested in a regression (see point 5). In addition, we aimed to show the "primary" relation by comparing the respective beta weights of the conspiracy theories and testing whether they*

	were significantly different from each other. This was done manually outside of the syntax based on instruction by Cohen et al.
**3) Describe the key dependent variable(s) specifying how they will be measured.**<br>List of COVID-related actions:<br>never<br>1 2 3 4 5 6 Always/ strongly<br>7<br>washing hands after being outside<br>not touching the face while being outside<br>disinfecting hands after being outside<br>wearing protective face masks out of the house<br>avoiding social contacts<br>staying at home in quarantine<br>stocking up on sanitary items<br>avoiding crowds<br>buying weapons for defense and security purposes<br>using alternative remedies like homeopathy or essential oils<br>buying equipment for water storage and water purification<br>withdrawing available cash from my bank account<br>invest in stock market<br>stocking up on petrol and oil<br>searching information by alternative media online<br>PCA with loadings > .30 on one and < .30 on the other factor will determine which items to keep in which scale.	*Variables were measured as described but instead of Principal Component Analyses we conducted Exploratory Factor Analyses (with promax rotation) as the more adequate method for extracting factors. This decision was not based on the data and both analyses lead to virtually identical results with PCA providing somewhat stronger factor loadings.*
In the UK only, compliance with new lockdown:<br>comply with the curfew rules +<br>go out to meet friends from time to time -<br>go directly home from work/ grocery shopping without seeing anyone +<br>briefly chat with friends/ neighbours when I meet them on the street -<br>hang out in groups of friends at private places -<br>hang out in groups of friends in public places -<br>Conspiracy theory 1:<br>The virus is intentionally presented as dangerous in order to mislead the public.<br>Experts intentionally mislead us for their own benefit, even though the virus is not worse than a flu.<br>We should believe experts when they say that the virus is dangerous.<br>Comnspiracy Theory 2:<br>Corona was intentionally brought into the world to reduce the population.<br>Dark forces want to use the virus to rule the world.<br>I think it's nonsense that the virus was created in a laboratory.	*Variables were measured as described:*<br><br>*Compliance with lockdown:*<br>`compute noncompl = mean(Q23_1r, Q23_3r, Q23_2, Q23_4, Q23_5, Q23_6).`<br><br>*Conspiracy Theory 1:*<br>`compute CT_hoax = mean (SpecCTs_1, SpecCTs_2, SpecCTs_3r).`<br><br>*Conspiracy Theory 2:*<br>`compute CT_weapon = mean (SpecCTs_4, SpecCTs_5, SpecCTs_6r).`
**5) Specify exactly which analyses you will conduct to examine the main question/hypothesis.**<br>Prediction of recommended actions (items selected based on PCA; expected to include hygiene and physical distance behavior) and non-recommended actions (same selection criteria; expected to include buying guns and stocking up on petrol and sanitary	*As mentioned above, EFA rather than PCA was used to extract factor structure (but PCA yielded identical results). Simultaneous prediction of behaviors by both CTs refers to Step 1 in the central regression analyses. Based on this step, beta weights of both are also compared.*

items, as well as alternative and homeopathic remedies) by simultaneously including both CTs.	`REGRESSION`<br>`  /MISSING LISTWISE`<br>`  /STATISTICS COEFF OUTS R ANOVA CHANGE`<br>`  /CRITERIA=PIN(.05) POUT(.10)`<br>`  /NOORIGIN`<br>`  /DEPENDENT action_reco action_non`<br>`  /METHOD=ENTER CT_weapon CT_hoax`<br>`/METHOD=STEPWISE p1 RWA SDO`<br>`/METHOD=ENTER B5O B5C B5E B5A B5N AFFECTED_cov age.`
**6) Describe exactly how outliers will be defined and handled, and your precise rule(s) for excluding observations.** People who recommend their data not be used will be deleted from the sample.	*Followed as planned.*<br>*In Syntax:*<br>`select if q80 GT 4.`<br>`execute.`
**7) How many observations will be collected or what will determine sample size? No need to justify decision, but be precise about exactly how the number will be determined**. N = 300 UK-based paerticipants via Prolifc; N = 300 US-based participants via MTurk.	*Done as planned.*
**8) Anything else you would like to pre-register? (e.g., secondary analyses, variables collected for exploratory purposes, unusual analyses planned?)** As control variables, we will also measure conspiracy mentality, political orientation, SDO, RWA, Big 5, the extent of being affected by COVID-19	*Steps 2 and 3 of the central regressions include these variables as additional control variables to rule out spurious correlations due to overlap with these. Not specified here, political orientation, RWA and SDO were entered in a stepwise procedure to avoid multicollinearity and resulting spurious suppression effects:*<br><br>`REGRESSION`<br>`  /MISSING LISTWISE`<br>`  /STATISTICS COEFF OUTS R ANOVA CHANGE`<br>`  /CRITERIA=PIN(.05) POUT(.10)`<br>`  /NOORIGIN`<br>`  /DEPENDENT action_reco action_non`<br>`  /METHOD=ENTER CT_weapon CT_hoax`<br>`/METHOD=STEPWISE p1 RWA SDO`<br>`/METHOD=ENTER B5O B5C B5E B5A B5N AFFECTED_cov age.`

Supplement 3: Detailed regression tables for Study 2a and 2b

Table.S2

Results of the Stepwise Regression Analysis in Study 2a (US) for recommended pandemic behavior

	Model 1				Model 2				Model 3			
Block of predictors	*B*	*SE*	β	*p*	*B*	*SE*	β	*p*	*B*	*SE*	β	*p*
Block 1: Conspiracy Theories												
COVID-19 Hoax	-.448	.052	-.601	< .001	-.473	.052	-.634	< .001	-.302	.052	-.405	< .001
SARS-Cov-2 Human-Made	.080	.052	.106	.129	.045	.053	.060	.395	-.009	.050	-.011	.863
Block 2: including political orientation												
Political Orientation					.098	.035	.158	.005	.066	.032	.107	.041
Right-Wing Authoritarianism (RWA)												
Social Dominance Orientation (SDO)												
Block 3: control variables												
COVID-19 Threat									.320	.042	.367	< .001
Openness (Big 5)									.031	.061	.025	.611
Conscientiousness (Big 5)									.287	.068	.224	.000
Extraversion (Big 5)									-.122	.058	-.103	.036
Agreeableness (Big 5)									.067	.062	.054	.287
Neuroticism (Big 5)									.019	.059	.017	.753
Age									.008	.005	.072	.131

Note. N = 288.

Table.S3

Results of the Stepwise Regression Analysis in Study 2a (US) for non-recommended pandemic behavior

	Model 1				Model 2				Model 3			
Block of predictors	*B*	*SE*	β	*p*	*B*	*SE*	β	*p*	*B*	*SE*	β	*p*
Block 1: Conspiracy Theories												
COVID-19 Hoax	.252	.062	.261	< .001	.188	.056	.195	.001	.254	.053	.263	< .001
SARS-Cov-2 Human-Made	.412	.062	.423	< .001	.282	.058	.290	< .001	.216	.052	.222	< .001
Block 2: including political orientation												
Political Orientation												
Right-Wing Authoritarianism (RWA)					.617	.077	.380	< .001	.318	.075	.196	< .001
Social Dominance Orientation (SDO)												
Block 3: control variables												
COVID-19 Threat									.406	.045	.359	< .001
Openness (Big 5)									-.054	.065	-.034	.408
Conscientiousness (Big 5)									-.211	.070	-.127	.003
Extraversion (Big 5)									.126	.060	.083	.037
Agreeableness (Big 5)									-.026	.065	-.016	.693
Neuroticism (Big 5)									-.063	.060	-.044	.301
Age									-.016	.005	-.118	.002

Note. *N* = 288.

Table.S4

Results of the Stepwise Regression Analysis in Study 2b (UK) for recommended pandemic behavior

	Model 1				Model 2				Model 3			
Block of predictors	*B*	*SE*	β	*p*	*B*	*SE*	β	*p*	*B*	*SE*	β	*p*
Block 1: Conspiracy Theories												
COVID-19 Hoax	-.196	.060	-.218	.001	-.172	.060	-.191	.005	-.082	.060	-.091	.178
SARS-Cov-2 Human-Made	.085	.045	.125	.060	.096	.045	.141	.034	.043	.044	.064	.324
Block 2: including political orientation												
Political Orientation					.148	.043	.239	.001	.149	.044	.240	.001
Right-Wing Authoritarianism (RWA)												
Social Dominance Orientation (SDO)					-.213	.057	-.263	< .001	-.220	.057	-.270	< .001
Block 3: control variables												
COVID-19 Threat									.190	.049	.226	< .001
Openness (Big 5)									.041	.055	.043	.453
Conscientiousness (Big 5)									.157	.069	.140	.023
Extraversion (Big 5)									.069	.055	.072	.206
Agreeableness (Big 5)									-.068	.062	-.064	.275
Neuroticism (Big 5)									-.096	.052	-.108	.067
Age									-.008	.004	-.113	.052

Note. *N* = 298.

Table.S5

Results of the Stepwise Regression Analysis in Study 2b (UK) for non-recommended pandemic behavior

	Model 1				Model 2				Model 3			
Block of predictors	*B*	*SE*	β	*p*	*B*	*SE*	β	*p*	*B*	*SE*	β	*p*
Block 1: Conspiracy Theories												
COVID-19 Hoax	.028	.046	.040	.538	-.004	.046	-.006	.922	.045	.048	.063	.346
SARS-Cov-2 Human-Made	.153	.035	.284	< .001	.138	.034	.256	< .001	.118	.034	.220	.001
Block 2: including political orientation												
Political Orientation												
Right-Wing Authoritarianism (RWA)												
Social Dominance Orientation (SDO)					.143	.037	.221	< .001	.137	.038	.212	< .001
Block 3: control variables												
COVID-19 Threat									.105	.039	.157	.008
Openness (Big 5)									.061	.043	.080	.155
Conscientiousness (Big 5)									.055	.054	.062	.302
Extraversion (Big 5)									.024	.043	.031	.581
Agreeableness (Big 5)									-.067	.049	-.079	.175
Neuroticism (Big 5)									-.075	.041	-.107	.069
Age									-.004	.003	-.075	.183

Note. *N* = 298.

Table.S6

Results of the Stepwise Regression Analysis in Study 2b (UK) for non-compliance with lockdown

	Model 1				Model 2				Model 3			
Block of predictors	*B*	*SE*	β	*p*	*B*	*SE*	β	*P*	*B*	*SE*	β	*p*
Block 1: Conspiracy Theories												
COVID-19 Hoax	.197	.039	.328	< .001	.182	.039	.302	< .001	.134	.041	.223	.001
SARS-Cov-2 Human-Made	-.046	.029	-.101	.120	-.053	.029	-.116	.074	-.028	.030	-.061	.350
Block 2: including political orientation												
Political Orientation												
Right-Wing Authoritarianism (RWA)												
Social Dominance Orientation (SDO)					.066	.032	.121	.037	.073	.033	.135	.026
Block 3: control variables												
COVID-19 Threat									-.099	.034	-.176	.004
Openness (Big 5)									.029	.037	.046	.425
Conscientiousness (Big 5)									-.081	.046	-.107	.083
Extraversion (Big 5)									.012	.037	.018	.750
Agreeableness (Big 5)									.050	.043	.070	.239
Neuroticism (Big 5)									-.047	.036	-.080	.183
Age									.003	.003	.053	.359

Note. $N = 298$.

Supplement 4: Supplemental Study in German context.

We had a chance to replicate our findings in the German context by including our two conspiracy scales as well as a few items related to prepping into the COVID-19 battery of the Social Cognition Center Cologne. Further studies that were run within the battery are reported elsewhere (Dohle et al., 2020; Dorrough et al., 2020; Glöcknet et al., 2020a, 2020b; Schneider & Dorrough, 2020). We pre-registered our analyses at https://aspredicted.org/jt43s.pdf.

Method

Participants

A total of *N*=301 participants were recruited in representative quotas for the German age distribution over the age of 18 and the gender distribution (see pre-registration for details). The final sample consisted of 143 men, 156 women; M_{age}=50.06, SD_{age}=16.15).

Measures

We translated the two conspiracy beliefs in a dual-forward way and resolved inconsistencies via a joint discussion. As an equivalent to the containment-related behavior we relied on a scale of “adoption of protective measures” already included in the project by other authors (Dohle, Wingen, & Schreiber, 2020). On this scale, participants indicated how frequently (from never to always; 5-points) they engaged in twelve behaviors in the domains of personal hygiene (washing hand with soap; sneeze or cough in the elbow; wear face masks or scarfs in public), and physical distancing (refrain from shaking hands and hugs; keep 6 feet distance in public; stay home as much as possible; work from home whenever possible; avoid rush hours in stores; avoid family gatherings; avoid crowds; avoid public transport; reduce personal meetings with ill or vulnerable people). It should be noted that in between the studies reported in the manuscript and this study, the public and expert opinion on the

usefulness of facemasks had shifted considerably, with them now being seen as instrumental in reducing the spread from an infected person. Prepping behavior was adapted to the German context and completed on the same scale. Specifically, participants indicated how frequent they hoarded emergency supplies, hoarded facemasks, shielded themselves off against 5G radiation, build up defense measures, and hoarded durable foods. These prepping items where embedded in filler items tapping into pro-social behavior (e.g., run errands for vulnerable neighbors) for which we pre-registered no hypotheses. Political orientation was assessed with scale from *left* (1) to *right* (10).

Results and Discussion

All scales proved sufficiently reliable (Table.S7). To test whether the data would support our prediction that hoax beliefs would negatively predict less containment-related behavior, but belief about human origin of SARS-Cov-2 would positively predict prepping behavior, we ran two multiple linear regressions with the two conspiracy beliefs and political orientation as predictors, and the two kinds of behaviors as respective outcomes.

Table.S7

Intercorrelations of the key variables in supplemental study

	M	*SD*	α	1.	2.	3.	4.
1. COVID-19 Hoax	2.10	1.12	.880				
2. SARS-Cov-2 Human-Made	2.24	1.03	.708	**.545**			
3. Containment-related behavior	4.34	0.63	.888	**-.473**	**-.300**		
4. Self-centered prepping behavior	1.92	0.85	.847	**.188**	**.316**	-.047	
5. Political Orientation	4.73	2.09	-	.093	.137	-.121	.110

Note. *N* = 301. Significant Correlations at Bonferroni-corrected .005 (≥ .162) printed in bold.

In line with our predictions, containment-related behavior was solely predicted by hoax beliefs, *B* = -0.246, *SE* = 0.034, β= -.438, *p* < .001, but neither human-made beliefs, *B* = -0.031, *SE* = 0.037, β= -.051, *p* = .401, nor political orientation, *B* = -0.022, *SE* = 0.015, β= -

.074, p = .152. Specifically, hoax beliefs were stronger predictors than human-made beliefs, $\Delta\beta$=.387, $t(297)$=6.62, p<.001. On the contrary, human-made beliefs predicted prepping behavior, B = 0.243, SE = 0.054, β= .296, p < .001. Hoax beliefs did not, B = 0.016, SE = 0.050, β= .021, p = .750, and neither did political orientation, B = 0.027, SE = 0.022, β= .076, p = .227. Human-made belief thus had a significantly stronger prediction than hoax beliefs, $\Delta\beta$=.275, $t(297)$=4.35, p<.001.

These analyses thus fully replicated the pattern reported in the paper in yet another context, with differently worded and contextually adapted behavioral indicators. This speaks to the robustness of the observed effect.

References

Dohle, S., Wingen, T. & Schreiber, M. (2020). Acceptance and adoption of protective measures during the Covid-19 pandemic: The role of trust in politics and trust in science. Manuscript in preparation.

Dorrough, A.R., Hellmann, D.M., & Glöckner, A., (2020). Social preferences in times of Corona: The role of responsibility on prosocial behavior. Manuscript in preparation.

Glöckner, A., Dorrough, A.R., Wingen, T., & Dohle, S. (2020a, May 10). The Perception of Infection Risks during the Early and Later Outbreak of COVID19 in Germany: Consequences and Recommendations. Manuscript in preparation.

Glöckner, A., Dorrough, A.R., Michels, M., & Giersch, D. (2020b, May 14). Personality, Dread and Adherence to Governmental Measures in the COVID19 pandemic. Manuscript in preparation.

Schneider, I.K., & Dorrough, A.R. (2020, May 14). The role of ambivalence in adherence to Covid-19 measures. Manuscript in preparation.
